# Light Readout of Small Scintillators Using SiPM Photosensors

**DOI:** 10.3390/s25206412

**Published:** 2025-10-17

**Authors:** Chiara Rita Failla, Simone Amaducci, Gaetano Elio Poma, Paolo Finocchiaro

**Affiliations:** 1 INFN-Laboratori Nazionali del Sud, Via S. Sofia 62, 95123 Catania, Italy; failla@lns.infn.it (C.R.F.); amaducci@lns.infn.it (S.A.); elio.poma@lns.infn.it (G.E.P.); 2 Department of Physics and Astronomy, University of Catania, Via S. Sofia 64, 95123 Catania, Italy

**Keywords:** silicon photomultiplier, scintillator, non-linearity

## Abstract

**Highlights:**

**What are the main findings?**

- A light readout from scintillators by means of a SiPM can or cannot give rise to non-linearity at a high energy, depending on the light decay time and on the number of SiPM microcells.

**What is the implication of the main finding?**

- A careful choice of scintillator and SiPM can prevent or minimize non-linear behavior at a high deposited energy.

**Abstract:**

During the last two decades, relevant progress has been achieved in silicon photomultiplier (SiPM) technology, such that in an increasing number of radiation detection applications they are proposed as a viable alternative to traditional photomultiplier tubes (PMTs). Applications where the light from tiny scintillating crystals is detected by a single SiPM raise the question of the possible non-linearity of the response due to the saturation of the number of microcells involved. In other cases, where larger scintillators subtend arrays of SiPMs, the same question could hold. This work tries to disentangle such a question with a realistic numerical approach and a few tests showing that the possible saturation effects depend on the interplay between the features of the scintillator and of the SiPM (array). The quantitative results of this analysis can likely be used to better plan future radiation detection systems and to highlight their linearity boundaries.

## 1. Introduction

The detection of gamma rays is one of the most widespread applications in radiation physics worldwide. Whenever high spectroscopic precision is required, in order to identify the emitting radioisotopes, the gold standard of detectors is the germanium semiconductor [1]. In most other cases, much less expensive and more practical detectors based on scintillators are exploited. A scintillator detector is based on special organic or inorganic materials exhibiting the notable feature of producing tiny flashes of light when hit by radiation. We will not go into detail on how such light is produced at a molecular level, but we will just remind the reader here that the produced visible photons are emitted isotropically according to an exponential time distribution with a decay constant µ characteristic of each material. Organic scintillators are typically faster, with µ values even down to 1–2 ns, whereas inorganic crystals span a wide range from tens of nanoseconds up to milliseconds [2,3].

Over several decades, the light readout of scintillators has mainly been performed by means of photomultiplier tubes (PMTs), until more recently a new kind of solid-state photodetector has been developed, namely the silicon photomultiplier (SiPM). Such a device, suggested many years ago but only recently industrially produced because of relevant technological advances, operates according to a quasi-digital scheme. Indeed, it consists of an array of semiconductor microcells kept in a quiescent state slightly above the breakdown voltage. When a visible photon interacts with one such microcell, it triggers a discharge, limited by a built-in quenching resistor, that gives rise to a signal consisting of the electrical charge stored in the microcell itself. All microcells being identical, each one produces the same signal when hit by a visible photon, and then a light pulse consisting of n photons should produce a signal n times as large as the single photon does.

Of course, this ideal scheme has several limitations in real sensors:

1. Only a fraction of the photons hitting the SiPM produce signals because of its photon detection efficiency (PDE), which is far from 100%;
2. When two or more photons interact with the same microcell, the produced signal is the same (this is the so-called problem of the multiple hit);
3. Each microcell has a recharging dead time, i.e., a short time interval after being triggered, during which it is inactive because its voltage is being restored to the operating value;
4. As the microcells are kept above breakdown, every now and then they can spontaneously discharge for thermal reasons, giving rise to an overall Poissonian dark noise mainly consisting of single microcell signals plus rarer double, triple and higher order spurious coincidences.

However, the main limitation of SiPMs is their maximum size, which for the currently available devices is 6 mm × 6 mm. Such a small size can also be seen as an advantage should one be interested in setting up a miniature gamma-ray detector. Due to the decreasing cost of these photosensors, the size limitation is currently being overcome by arranging arrays of SiPMs for the readout of larger scintillators. Details on the features and operation of SiPMs can be found in [4,5,6,7] and in the copious list of references therein.

One of the questions arising from the above-mentioned operational limitations is the linearity of the response, in particular, when detecting gamma rays of the order of 1–2 MeV [8,9,10,11]. In this work, we examine the behavior of two different models of SiPM (Table 1) when coupled to five popular scintillators (Table 2) as a function of the energy deposited by gamma rays. In Section 2, we describe the algorithm developed to evaluate the behavior of a SiPM illuminated by scintillation light; in Section 3, the model is applied to a SiPM detecting light from scintillators; and, in Section 4, we show the results obtained with the several examined combinations of scintillator + SiPM, along with the experimental results obtained in two particular real cases. Finally, in Section 5, we discuss the relevant take-home messages arising from our results.

**Table 1:** Main features, relevant to this study, of the two selected SiPMs.

	MICROFC−60035−SMT SensL (Now OnSemi) [12]	S14160-6050HS Hamamatsu [13]
Number of microcells	18,980	14,331
Microcell recharge time [ns]	100	92

**Table 2:** Main features, relevant to this study, of the selected five popular scintillators.

	CsI(Tl)	LaBr3(Ce)	CeBr3	BGO	NaI(Tl)
Light yield [photons/keV]	60	70	70	10	45
Decay time [ns]	960	30	20	300	250
Emission spectrum from ref.	[14]	[15]	[16]	[17]	[18]
Refractive index at *λ* max	1.8	1.9	2.1	2.1	1.8
Weighted PDE SensL [%]	27%	31%	34%	29%	35%
Weighted PDE Hamamatsu [%]	41%	42%	45%	44%	48%

## 2. Modeling the SiPM Response to Light

### 2.1. Simulation Procedure

There are two different mechanisms possibly leading to the loss of linearity in a SiPM: (i) the signal saturation due to multiple photons interacting with the same microcell but being detected as one; and (ii) the loss of photons due to microcells being hit during their recharge dead time because of a previous hit. The number of microcells actually fired by a bunch of photons can be smaller than the number of electron avalanches generated in the SiPM, since two or more avalanches generated in the same microcell produce the same signal and thus are seen as one. The most probable number of microcells *f*(*q*) actually triggered (i.e., when a photoelectron is produced capable of producing an avalanche) by a bunch of *q* photons impinging randomly on a SiPM with *m* microcells and PDE equal to *p* can be evaluated based on the binomial distribution.

- $\frac{p}{m}$ is the probability of a single microcell being triggered by one impinging photon;
- $\left(1-\frac{p}{m}\right)$ is the probability that a single microcell is not triggered by one impinging photon;
- ${\left(1-\frac{p}{m}\right)}^{q}$ is the probability that a single microcell is not triggered by *q* impinging photons;
- $\left[1-{\left(1-\frac{p}{m}\right)}^{q}\right]$ is the probability that a single microcell is triggered by at least one of the q impinging photons.

As there are *m* microcells, the expected number of triggered ones is calculated as follows:

(1) $$f\left(q\right)=m\left[1-{\left(1-\frac{p}{m}\right)}^{q}\right]$$

By exploiting the notable limit shown below

(2) $$\underset{n\to \infty }{\lim}{\left(1+\frac{x}{n}\right)}^{n}=e^{x}$$

one obtains the much simpler approximate formula:

(3) $$f\left(q\right)=m\left(1-e^{-\frac{pq}{m}}\right)$$

The effect of the possible loss due to the dead time of recharging microcells requires a consideration of the time development of the light pulse. Therefore, we developed a simple algorithm that considers the production of photons exponentially decreasing with time, with the decay constant of the scintillator and using time slots of 5 ns. In each time slot, the number of triggered microcells is calculated by means of Equation (3), and decreased by the number of hits occurring in microcells currently recovering because they were previously triggered and are temporarily unavailable. In reality, during the recovery, there could be a partial signal, whose amplitude grows slowly by a (1 − exp^−t/τ^) function, only significant in the final stage of the recharge, i.e., in the short time it takes the microcell bias to go from the breakdown voltage to overvoltage. Indeed, below the breakdown, no signal is produced. Moreover, the information from the manufacturers only states a stark number for the recovery time, not specifying the relationship with τ. Therefore, we opted for a stepwise yes/no approach, with the recovering microcells switched off during the recovery time, confident that this does not make a relevant difference in the overall behavior, even though it can give rise to some sharper structures in the plots. The algorithm follows the light pulses over a few microseconds, which is much longer than the slowest decay constant of the considered scintillators, and calculates the sum of all triggered microcells, which is proportional to the integral of the output signal.

### 2.2. Cross-Check with an Empirical Formula

In Ref. [19], Grodzicka et al. proposed an empirical formula (Equation (4)) in order to take care of the recharge time of the microcells, the additional hits produced by afterpulses and cross-talk and the light pulse duration. The formula was used by the authors to fit the experimental data produced by means of light pulses into a controlled environment.

(4) $$f\left(q\right)=m\left(\frac{P_{w}}{t_{d}}\right)\left(1-e^{-\frac{pq\left(1+P\right)}{m\left(\frac{P_{w}}{t_{d}}\right)}}\right),\;P_{w}>t_{d}$$

The new variables with respect to Equation (3) are *P*, which represents the fraction of additional hits due to afterpulses and cross-talk, *P_w_*, which is the input light pulse duration, and *t_d_*, which is the microcell recovery time. We remark that the formula implicitly assumes a yes/no approach for the microcell recovery process. We used Equation (4) to calculate the expected number of hits as a function of the number of ideal hits *pq* (i.e., if no counting loss due to multiple hits was present). The parameters were those of the S10362-33-050C SiPM listed in the article, namely 3600 microcells with a 50 ns recovery time.

**Figure 1:**
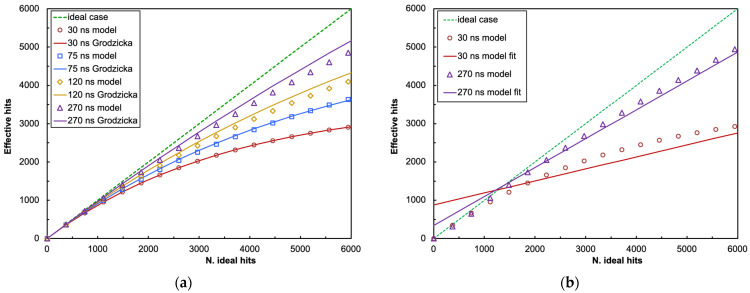
(**a**) Predictions of our model under the same conditions of Ref. [19]. (**b**) Two examples illustrating how a linear fit does not pass through the origin anymore because of the counting losses.

We then tuned our model in order to simulate the same SiPM hit by trapezoidal light pulses similar to those employed in Ref. [19], where they were produced by an LED, driven by a pulse generator featuring a 5 ns rise-and-fall slope that excited a very fast plastic scintillator whose light was finally detected by the SiPM. In the model, we used a 1 ns time step to better cope with very short pulses, even though we did not see an appreciable difference when using a 5 ns time step. The authors of Ref. [19] did not specify the *p* value, so we decided to neglect it both in the formula and in our model, as it is of the order of a couple of percent at a normal operating overvoltage [12,13]. The resulting data, plotted in Figure 1a, show that the predictions of our model are in reasonable agreement with Ref. [19], even though it yields fewer expected hits as compared to the empirical formula. This is likely due to our model finely following the time evolution of the light pulse detection, with hit and recovery of the microcells. We remark that, despite the non-linearity of the SiPM behavior, because of the counting loss deriving from Equation (3), a linear fit in several cases can still reasonably follow the data, depending on the number of photons successfully hitting the SiPM and the pulse duration. However, the price to pay is the need for a compensating offset term, and the linear fit does not pass anymore through the origin, as illustrated in the two examples in Figure 1b.

**Figure 2:**
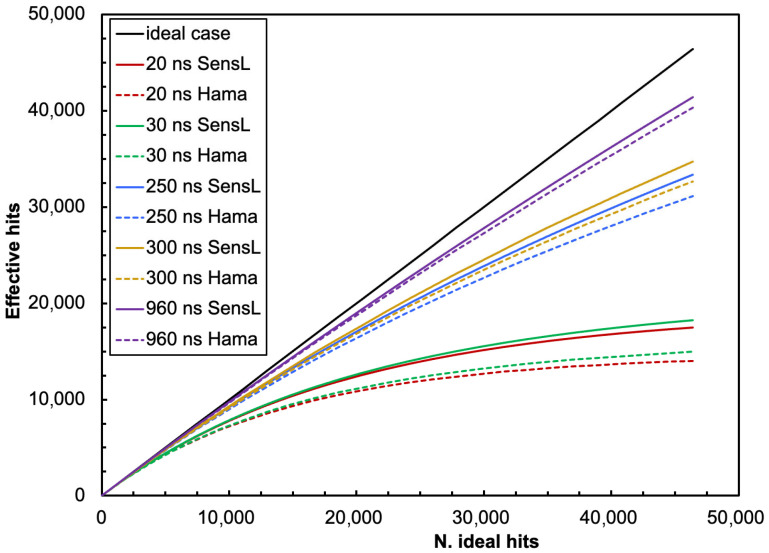
Predictions of our model with the two SiPMs under study solicited by exponential light pulses, with the decay constants of the scintillators listed in Table 2.

Confident that our SiPM model can reasonably reproduce the experimental data, we used it to calculate the number of effective hits as a function of the number of ideal ones when our two SiPMs under study are solicited by exponential light pulses, with the decay constants of the scintillators listed in Table 2. In order to further explore the SiPM behavior in terms of the energy deposited into the crystals, the investigated range of ideal hits in the formula was conveniently extended. The results, shown in Figure 2, clearly indicate that, the slower the light pulse, the closer the SiPM response is to the ideal case.

## 3. Modeling Gamma-Ray Detection with Scintillation Detectors

### 3.1. Detecting Gamma Rays

The interaction of gamma rays with matter occurs according to three main processes:

- The photoelectric effect, dominating at a low energy, when the gamma disappears, transferring all of its energy to an electron;
- Compton scattering, dominating at an intermediate energy, with the gamma scattering off an electron and imparting it with some kinetic energy;
- e+e− pair creation close to a nucleus, exploiting 1.022 MeV of the incoming gamma and thus being the dominating effect at a very high energy.

For the low-to-medium energy range considered here, namely up to 2.5 MeV, only the photoelectric effect and Compton scattering are relevant. The energetic recoil electron produces ionizations by colliding with other electrons, which are freed and whose total kinetic energy is equal to the energy released by the initial gamma interaction. The scintillation light in scintillators is produced by the interaction of these electrons with “color centers”, i.e., typically dopants in the material capable of reaching an excited level through collision, which deexcites them by emitting visible light. The number of visible photons produced in each gamma interaction is generally proportional to the energy deposited by the gamma ray into the material. The detection and energy measurement of the gamma rays is achieved by measuring the amount of scintillation light produced by means of some photodetector, which converts light into an electric signal. Due to the very small amount of light produced, a physical amplification is quite often required, and the task is accomplished by using a photomultiplier device.

Different scintillation detectors have a different linearity between the deposited energy and scintillation light, especially at a high deposited energy where some saturation of the light yield could be expected. Saturation can also be expected due to the employed photosensor, especially in the case of photoelectric interactions when the full gamma energy is transferred to the material. Starting from our previous experience with detectors based on scintillators and SiPMs [20,21,22,23], we examined the five popular scintillation materials, whose main features relevant to this study are listed in Table 2 [24], and their response when coupled to two models of 6 mm × 6 mm SiPM [12,13], whose features are listed in Table 1.

### 3.2. Collecting the Scintillation Light

The number of scintillation photons collected on the photosensor depends on the geometric features of the detector and, more importantly, on the type and quality of the outer surface of the scintillator. Indeed, a bare scintillator would lose most of the light through its outer faces, basically collecting only those photons traveling straight from the emission point to the photosensor. This is why scintillators are generally coated with a highly reflective layer (paint, resin, …) to maximize the light collection efficiency, allowing photons to be collected also after several internal reflections. The reflector is not specular, but is white so that, contrary to the case of geometrical reflection, the light path inside the scintillator is quickly randomized. This way, any possible variation in the light collection efficiency with its emission position inside the scintillator is minimized. The typical reflectivity values of the employed reflector materials range from 0.9 to 0.96.

**Figure 3:**
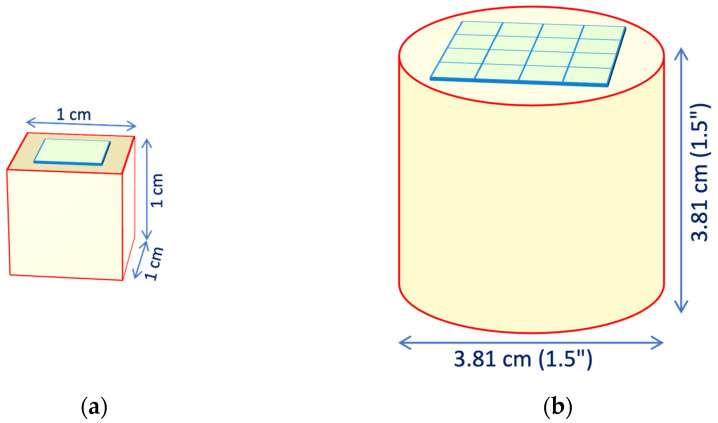
The two detector geometries studied: (**a**) 1 cm × 1 cm × 1 cm scintillator coupled to a single 6 mm × 6 mm SiPM; (**b**) cylindrical scintillator of 3.81 cm diameter and 3.81 cm height coupled to a square array of 4 × 4 SiPMs.

Two detector geometries have been examined, representative of two major possible approaches to the spectroscopic detection of gamma rays with scintillators and SiPMs. The first one concerns applications such as miniature detectors and dosimeters, whereas the second has a crystal size and shape typical of several existing commercial products:

- A compact configuration with a 1 cm × 1 cm × 1 cm scintillator coupled to a single 6 mm × 6 mm SiPM (Figure 3a);
- A bigger one with a cylindrical scintillator of 3.81 cm diameter and 3.81 cm height (1.5″ × 1.5″) coupled to a square array of 4 × 4 SiPMs (Figure 3b).

The results and considerations that we are going to describe in the following can be easily rescaled to similar geometrical configurations with larger or smaller scintillators and a different number of SiPMs.

Due to the compact size of both geometries with respect to the (re)absorption length of several tens of centimeters for all of the scintillators under consideration, in this study, we decided to neglect the possible self-absorption of scintillation photons in the scintillator material. The light collection efficiency, i.e., the fraction of photons reaching the SiPM, was first estimated by means of a simple naive approach:

- No real geometry is considered for the system, and no light propagation is implemented;
- A scintillation photon produced somewhere inside the crystal reaches a point on the inner surface;
- We assume it can hit the SiPM with a probability *ε* equal to the ratio between the area of the SiPM and the total area of the crystal;
- Otherwise, it can be reflected or absorbed with probability *r* and (1 − *r*), respectively, r being the reflectivity of the inner surface;
- We denote with *P*_1_ = *ε* the probability that the photon is collected directly on the first step, with *P*_2_ being the probability that the photon is collected after one reflection (i.e., at the second step), and so on;
- After each step, the probability of the photon being still available is (1 − *ε*)*r* (i.e., not collected and reflected), whereas the probability of being collected at the following step is still *ε*;
- The sum of all the probabilities of collection in any number of steps, regardless of the number of reflections, represents the light collection efficiency (Equation (5)).

This calculation was conducted for several values of reflectivity, ranging from 0.9 to 1, with the elementary collection probabilities *ε* = 0.06 and *ε* = 0.084 given by the area ratios for the cases of Figure 3a,b.

(5) $$P_{1}=\varepsilon \;P_{2}=\varepsilon \left(1-\varepsilon \right)r\;P_{3}=\varepsilon {\left[\left(1-\varepsilon \right)r\right]}^{2}\;\ldots \;P_{n}=\varepsilon {\left[\left(1-\varepsilon \right)r\right]}^{n-1}\;P=\underset{n}{\sum }P_{n}=\underset{n}{\sum }\varepsilon {\left[\left(1-\varepsilon \right)r\right]}^{n-1}=\varepsilon \frac{1-{\left[\left(1-\varepsilon \right)r\right]}^{n}}{1-\left(1-\varepsilon \right)r}\approx \frac{\varepsilon }{1-r+r\varepsilon }$$

In order to support or disprove these results, we performed a set of more sophisticated Monte Carlo simulation runs by means of Geant4 [25]. The inner surfaces of the scintillator were assumed to produce diffuse Lambertian reflection [26], each run with a different value of the reflectivity. Inside the crystal, we generated randomly 10^5^ scintillation photons per run, following each one throughout its path and reflections until being absorbed in a wall or reaching the photosensor. The two geometries of Figure 3 were implemented, and eleven runs per configuration were performed, with the reflectivity of the walls ranging from 0.9 to 1 in steps of 0.1. The runs with a reflectivity equal to one showed that the average times for the detection are 0.62 ns and 1.6 ns, respectively, which, assuming a refractive index around 1.9, correspond to average path lengths of the order of 10 and 25 cm. In the case of reflectivity equal to 0.95, these values roughly halve to about 5.5 and 13 cm, much smaller than the attenuation length values in the considered crystals, thus justifying the choice of neglecting the self-absorption.

**Figure 4:**
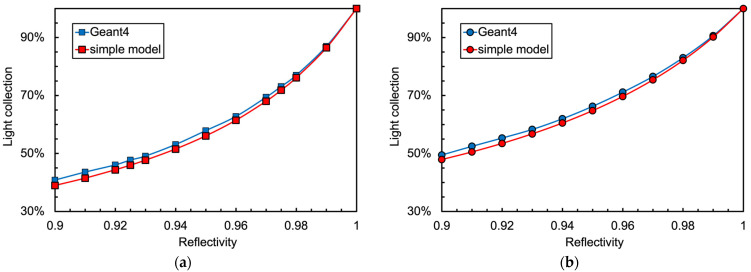
Simulation of the light collection efficiency as a function of the reflectivity of the walls: (**a**) for the cubic configuration of Figure 3a; (**b**) for the cylindrical configuration of Figure 3b.

**Figure 5:**
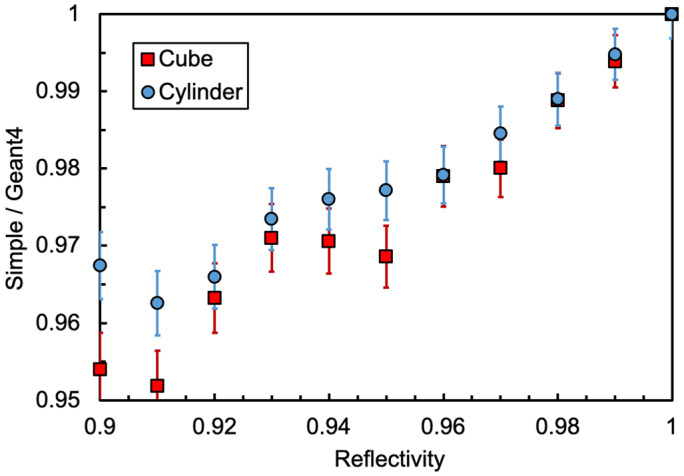
Ratio of simple-to-Geant4 between the curves of Figure 4. The quoted error bars are statistical from Geant4 hits.

The resulting values of the light collection efficiency for the two approaches and for the two detector geometries are plotted in Figure 4. Surprisingly, the differences between the simple and the Monte Carlo approaches are quite small, thus suggesting that the simple formula of Equation (5) can realistically be used for future evaluations of similar configurations. The ratio between the simple and Geant4 values is plotted in Figure 5 for the cube and the cylinder geometries, with the statistical error bars calculated from Geant4 hits. The data in Figure 4 and Figure 5 are also listed in Table A1 and Table A2 of Appendix A. For all of the following calculations, we used the light collection efficiency values of *p* = 0.56 and *p* = 0.65 resulting from Equation (5) for cube and cylinder geometries, respectively, choosing a reflectivity value of *r* = 0.95 and the above-mentioned elementary collection probabilities *ε* = 0.06 and *ε* = 0.084.

### 3.3. Detecting the Collected Light

**Figure 6:**
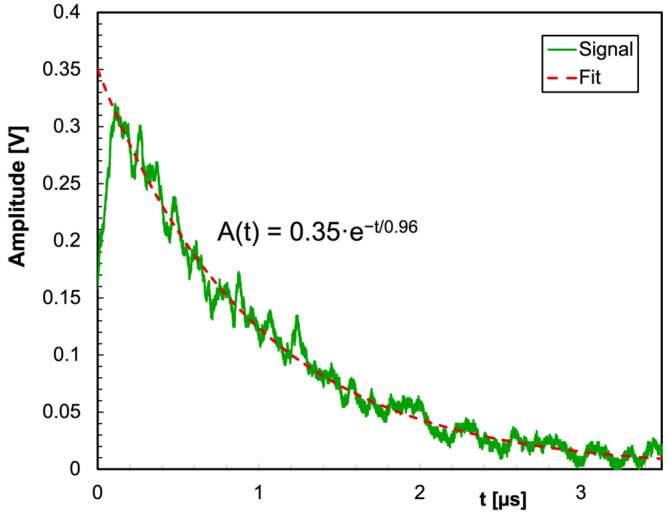
Example of a signal waveform acquired from a CsI(Tl) scintillator coupled to a SensL SiPM in the configuration of Figure 3a with a digital scope. The exponential fit to the waveform has a decay constant of 0.96 µs as expected.

The physical quantities to be considered for the detection with SiPMs are the light yield of the crystal, the light decay time, the emission spectrum, the collection efficiency of the chosen detector geometry, the SiPM’s PDE, its number of microcells and the microcell recovery time (i.e., dead time). If we want to calculate the expected response of the photosensor, we have to take into account all these quantities at the same time. We used the values listed in Table 1 and Table 2, and, in order to show the behavior of the decay time in real operation, we plotted in Figure 6 a signal waveform acquired from a CsI(Tl) scintillator coupled to a SensL SiPM in the configuration of Figure 3a with a digital scope. An exponential fit to the waveform produces a decay constant of µ = 0.96 µs as expected.

**Figure 7:**
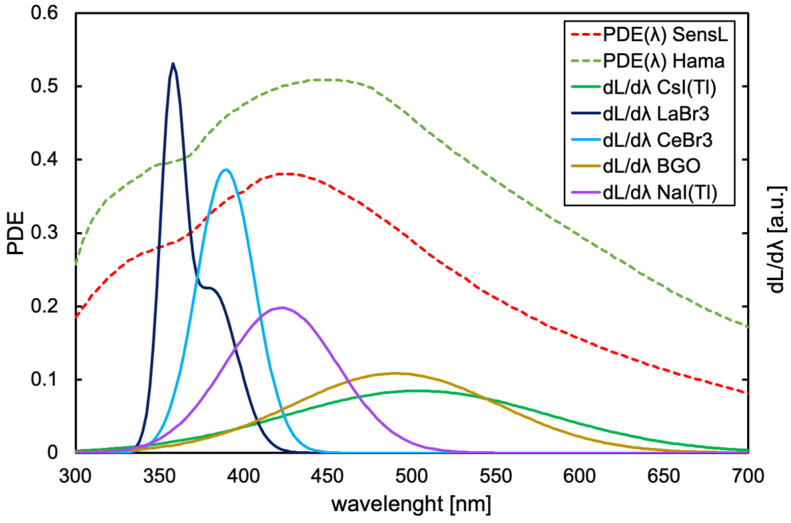
The photon detection efficiency of the two SiPMs [12,13] (left scale) and the light emission spectra of the five scintillators normalized to unit area [14,15,16,17,18] (right scale).

**Figure 8:**
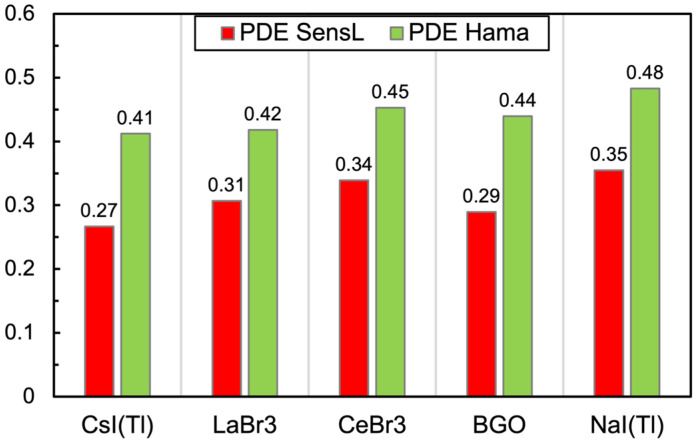
Effective PDE of the two SiPMs when detecting the light emitted by each scintillator, calculated by integrating the product of the PDE(λ) function with the light emission spectrum.

As for the PDE, we multiplied the PDE(λ) function of each SiPM [12,13] by the light emission spectra of the five scintillators [14,15,16,17,18] (Figure 7), normalized to the unit area. The integral of such a convolution represents the effective PDE of each SiPM when detecting the light emitted by each scintillator. The resulting values are reported in Figure 8. Obviously, the light collection also depends on the optical coupling between the crystal and the SiPM, which cannot be easily reproduced and controlled numerically. We decided to assume a perfect coupling.

## 4. Results

**Figure 9:**
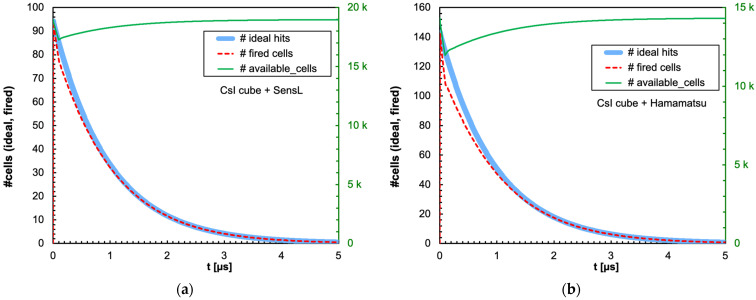
Time evolution of the number of triggered (dashed red line) and available (green line, right-hand scale) microcells, for the case of 2 MeV deposited in a 1 cm × 1 cm × 1 cm CsI(Tl) crystal. Also shown is the ideal number of microcells (light blue line) that would be triggered if no counting loss was present. (**a**) The case of the SensL SiPM. (**b**) The case of the Hamamatsu SiPM.

**Figure 10:**
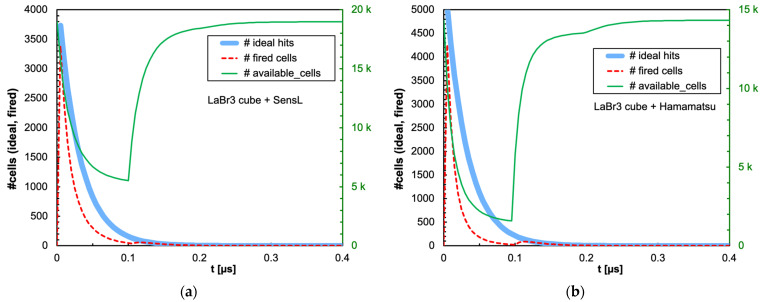
Time evolution of the number of triggered (dashed red line) and available (green line, right-hand scale) microcells, for the case of 2 MeV deposited in a 1 cm × 1 cm × 1 cm LaBr3(Ce) crystal. Also shown is the ideal number of microcells (light blue line) that would be triggered if no counting loss was present. (**a**) The case of the SensL SiPM. (**b**) The case of the Hamamatsu SiPM.

**Figure 11:**
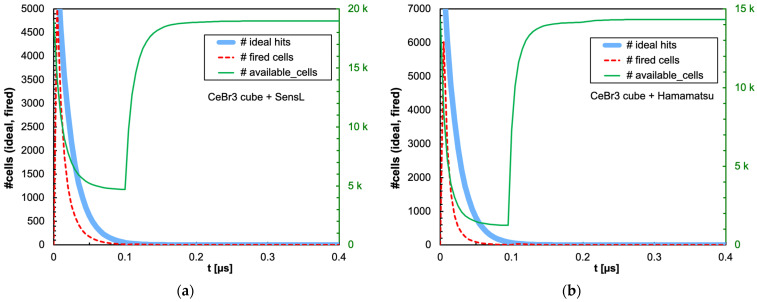
Time evolution of the number of triggered (dashed red line) and available (green line, right-hand scale) microcells, for the case of 2 MeV deposited in a 1 cm × 1 cm × 1 cm CeBr3 crystal. Also shown is the ideal number of microcells (light blue line) that would be triggered if no counting loss was present. (**a**) The case of the SensL SiPM. (**b**) The case of the Hamamatsu SiPM.

**Figure 12:**
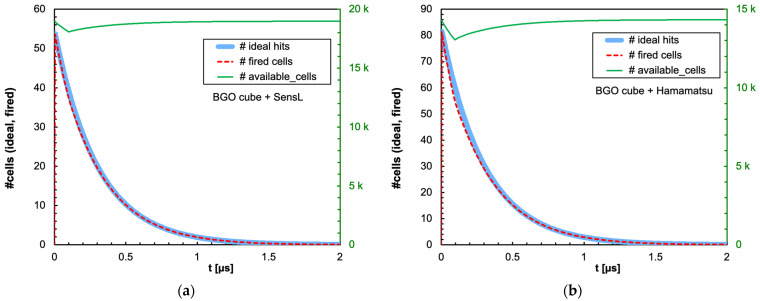
Time evolution of the number of triggered (dashed red line) and available (green line, right-hand scale) microcells, for the case of 2 MeV deposited in a 1 cm × 1 cm × 1 cm BGO crystal. Also shown is the ideal number of microcells (light blue line) that would be triggered if no counting loss was present. (**a**) The case of the SensL SiPM. (**b**) The case of the Hamamatsu SiPM.

**Figure 13:**
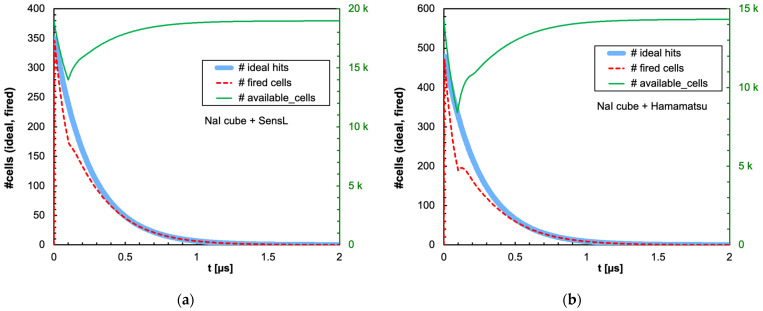
Time evolution of the number of triggered (dashed red line) and available (green line, right-hand scale) microcells, for the case of 2 MeV deposited in a 1 cm × 1 cm × 1 cm NaI(Tl) crystal. Also shown is the ideal number of microcells (light blue line) that would be triggered if no counting loss was present. (**a**) The case of the SensL SiPM. (**b**) The case of the Hamamatsu SiPM.

**Figure 14:**
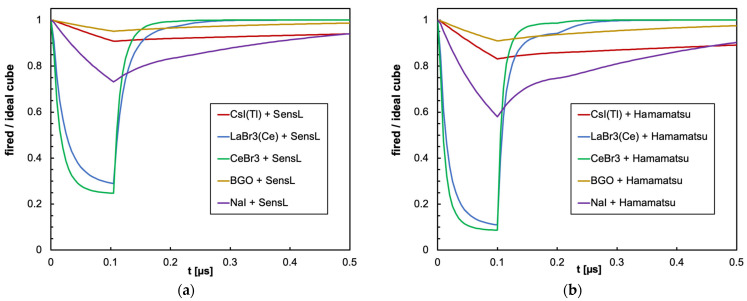
Summary of the ratio of the fired-to-ideal number of microcells as a function of time for the case of 2 MeV deposited in a 1 cm × 1 cm × 1 cm scintillator. (**a**) The case of the SensL SiPM. (**b**) The case of the Hamamatsu SiPM.

The combination of the light decay constant of the scintillator with the microcell recharge time of the SiPM produces an initial decrease in the number of available microcells that could or could not significantly influence the linearity of the SiPM response depending on the deposited energy, on the scintillator type and on the total number of microcells. In Figure 9a,b, we show as an example the number of triggered and of available microcells as a function of time, for the case of 2 MeV deposited in a 1 cm × 1 cm × 1 cm CsI(Tl) crystal read by the SensL and Hamamatsu SiPMs, respectively. Also shown is the ideal number of microcells that would be triggered if no recharge dead time were present. Figure 10, Figure 11, Figure 12 and Figure 13 show the corresponding plots for all the other combinations of SiPMs and scintillators listed in Table 1 and Table 2, respectively. These plots are useful for understanding the behavior of the SiPM during the development of the light pulse. It can be immediately observed that LaBr3(Ce) and CeBr3 give rise to a relevant counting loss due to the large amount of scintillation photons reaching the SiPM in a very short time interval, thus almost blinding it for a while and losing a considerable fraction of the light signal. This effect is more pronounced with the Hamamatsu SiPM due to its smaller number of microcells. Figure 14 summarizes the ratio of the fired-to-ideal number of microcells as a function of time for the five scintillators and the two SiPMs. Such a ratio is mainly determined by the number of available microcells at each instant, as it is evident from the shape of the curves as compared to the green curves in Figure 9, Figure 10, Figure 11, Figure 12 and Figure 13. We remark that the vertical scale starts from zero, thus highlighting how the fast LaBr3(Ce) and CeBr3 scintillators almost completely blind the SiPM at the beginning of the light pulse, more heavily for the Hamamatsu than for the SensL due to the higher PDE and the lower number of microcells.

**Figure 15:**
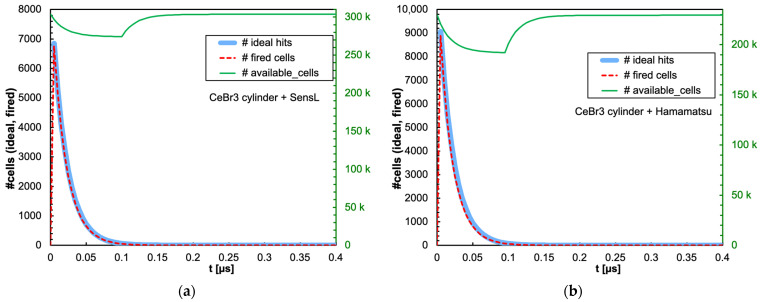
Time evolution of the number of triggered (dashed red line) and available (green line, right-hand scale) microcells, for the case of 2 MeV deposited in a 3.81 cm × 3.81 cm cylindrical CeBr3 crystal. Also shown is the ideal number of microcells (light blue line) that would be triggered if no counting loss was present. (**a**) The case of the SensL SiPM. (**b**) The case of the Hamamatsu SiPM.

**Figure 16:**
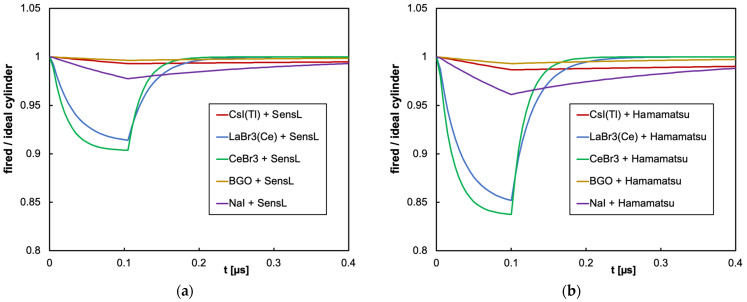
Summary of the ratio of the fired-to-ideal number of microcells as a function of time for the case of 2 MeV deposited in a 3.81 cm × 3.81 cm cylindrical scintillator. (**a**) The case of the SensL SiPM. (**b**) The case of the Hamamatsu SiPM.

As for the cylindrical configuration, we only show the time evolution plots for the worst case, which is the CeBr3 scintillator that features the highest light yield and the fastest decay time, in Figure 15a and b, respectively, for the SensL and Hamamatsu SiPMs. The ratio of the fired-to-ideal number of microcells as a function of time for the five scintillators and the two SiPMs in this configuration is shown in Figure 16. We remark that, in this case, no relevant SiPM blinding occurs (notice that the vertical scale starts from 0.8), thus indicating how a bigger crystal coupled to 16 SiPMs, featuring 16× microcells, strongly reduces the counting losses.

**Figure 17:**
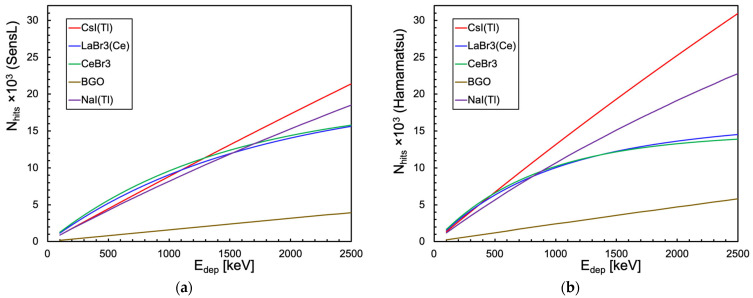
Calculation of the total number of triggered microcells as a function of the gamma energy deposited inside a cubic scintillator of Figure 3a. (**a**) When coupled to a SensL SiPM. (**b**) When coupled to a Hamamatsu SiPM.

**Figure 18:**
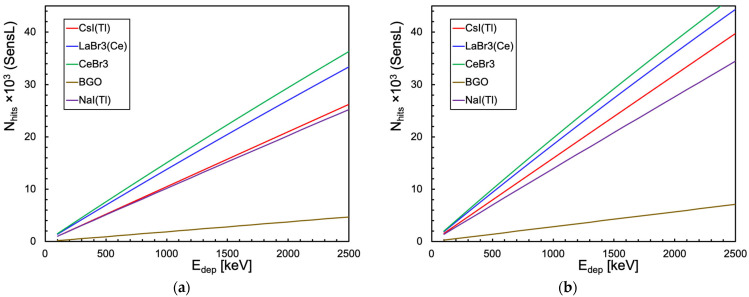
Calculation of the total number of triggered microcells as a function of the gamma energy deposited inside a cylindrical scintillator of Figure 3b. (**a**) When coupled to an array of 16 SensL SiPMs. (**b**) When coupled to an array of 16 Hamamatsu SiPMs.

**Figure 19:**
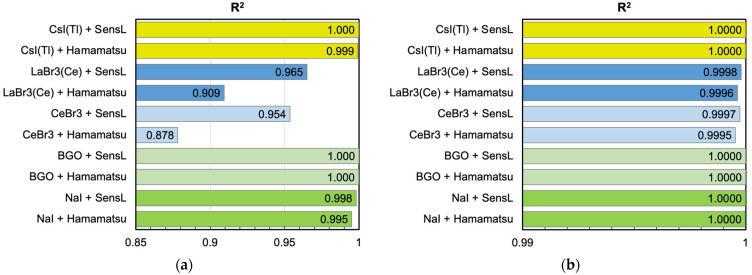
The *R*^2^ coefficient (Equation (6)), which quantifies the goodness of a linear fit, for all the scintillator + SiPM setups. (**a**) 1 cm × 1 cm × 1 cm crystal and one SiPM. (**b**) 3.81 cm × 3.81 cm cylindrical crystal and 16 SiPMs.

Then, we also calculated the response of each detector configuration as a function of the deposited gamma energy in the typical range up to 2.5 MeV. Figure 17 shows how a single SiPM coupled to small cubes of CsI(Tl), NaI(Tl) and BGO behave almost linearly, whereas when coupled to LaBr3(Ce) and CeBr3 they have a strongly non-linear behavior. The case of the bigger cylindrical scintillator coupled to 16 SiPMs, plotted in Figure 18, shows a much better behavior, apparently perfectly linear for all the combinations. The values reported in the four plots are listed in Table A3, Table A4, Table A5 and Table A6 of Appendix B. In order to numerically evaluate and compare the goodness of the linearity, we made a linear fit for each curve of Figure 17 and Figure 18 and then calculated the respective coefficient of determination (*R*^2^) values according to Equation (6).

(6) $$R^{2}=1-\frac{\sum_{i}{\left(y_{i}-y_{i}^{fit}\right)}^{2}}{\sum_{i}{\left(y_{i}-\bar{y}\right)}^{2}}$$

where *y_i_* is the number of hits produced by the model, $y_{i}^{fit}$ is the corresponding value obtained by the linear fit and $\bar{y}$ is the average of the *y_i_* values, with 0 ≤ *R*^2^ ≤ 1 and *R*^2^ = 1 indicating a perfect linear behavior. Figure 19a,b summarize the found *R*^2^ values for each detector setup, for cubic and cylindrical configurations, respectively. The considerations that were performed by looking at Figure 17 and Figure 18 find numerical support here.

**Figure 20:**
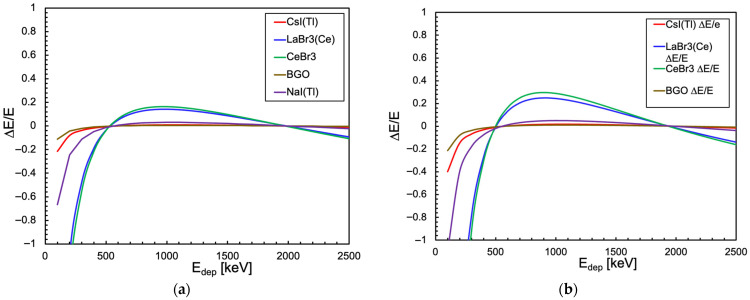
Relative difference between the expected energy value from the linear fit and its real value in the case of the cubic scintillator. (**a**) When coupled to a SensL SiPM. (**b**) When coupled to a Hamamatsu SiPM.

**Figure 21:**
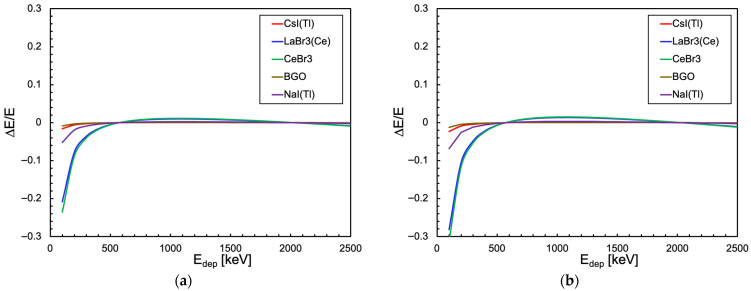
Relative difference between the expected energy value from the linear fit and its real value in the case of the cylindrical scintillator. (**a**) When coupled to an array of 16 SensL SiPMs. (**b**) When coupled to an array of 16 Hamamatsu SiPMs.

By inverting the linear fit, we can reconstruct the expected new energy scale and compare it with the true original one. In Figure 20, we plotted the relative difference between the expected energy value from the linear fit and its true value for the SensL and the Hamamatsu SiPMs in the case of the cubic scintillator. The plots represent the relative displacement (distortion) of each energy value reconstructed by a linear fit with respect to the true one. The corresponding plots for the cylindrical configuration are shown in Figure 21. We remark that the heavy distortion at a low energy is caused by the presence of an offset in the linear fit, necessary because of the form of Equation (3) (as already shown in Figure 1).

**Figure 22:**
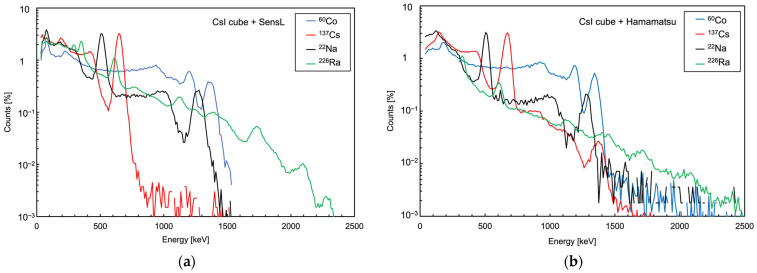
Spectra of four gamma sources acquired using a 1 cm × 1 cm × 1 cm CsI(Tl) crystal and a SiPM. (**a**) Crystal coupled to a SensL SiPM. (**b**) Crystal coupled to a Hamamatsu SiPM.

**Figure 23:**
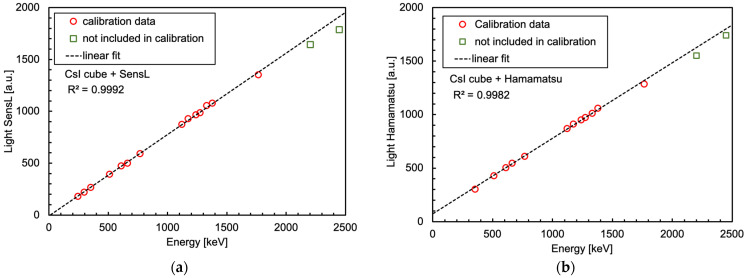
Calibration plots of the spectra shown in Figure 22. (**a**) SensL SiPM. (**b**) Hamamatsu SiPM.

**Figure 24:**
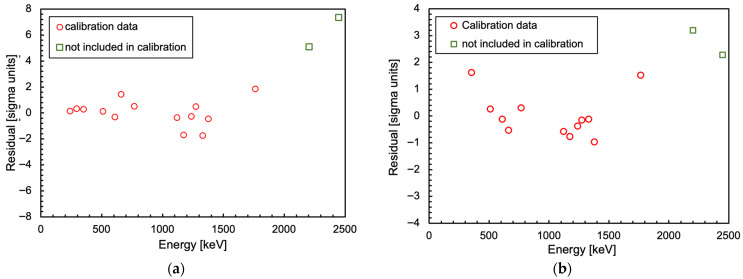
Normalized residuals of the calibration plots shown in Figure 23. (**a**) SensL SiPM. (**b**) Hamamatsu SiPM.

In Figure 22, we show two sets of spectra, taken with 1 cm × 1 cm × 1 cm CsI(Tl) crystals, read by a SensL and by a Hamamatsu SiPM, respectively. Each spectrum is related to one of four gamma sources (^226^Ra, ^22^Na, ^137^Cs, ^60^Co). The data were available from previous experiments, and unfortunately they were taken at different times using different sets of sources with different activities. This is why each spectrum was normalized to its own integral. The employed readout electronics were different as well; however, all the spectra were built by acquiring the waveforms, subtracting the baseline and numerically integrating the signal area (i.e., proportionally to the light detected by the SiPM) event by event. The spectra with the Hamamatsu SiPM were acquired in a noisier electronic environment and using lower activity radioactive sources, in particular, the ^226^Ra one. This is why two peaks, at 242 and 295 keV, could not be used, while the one at 352 keV was included in the following analysis with some doubts. Nonetheless, despite the very different experimental conditions in the two cases, the position of several energy peaks could be easily determined for the calibration. We selected the peaks up to 1764 keV (see Table 3) and performed the energy calibration by calculating a linear fit between the known energies and the observed peak positions in the spectra. The corresponding calibration plots are shown in Figure 23 and feature good *R*^2^ coefficient values of 0.9992 and 0.9982 for SensL and Hamamatsu, respectively. The normalized residuals of these two fits are plotted in Figure 24. Table 3 lists the energy values of the peaks used in the calibration for each source. The two highest energy peaks, from ^226^Ra, were not used for the calibration but just for a linearity cross-check. Indeed, the points at these two energies in Figure 23 and Figure 24 hint at a similar worsening linearity for both detectors.

## 5. Discussion

The main indication from our results is that, when planning to set up a spectroscopic gamma-ray detector based on a scintillator and SiPMs, careful consideration should be given both to the choice of components and to the energy range of interest. Indeed, there is a relevant interplay between the light yield of the scintillator, its decay time, the number of microcells featured by the SiPM and the microcell recovery time. The SiPM, as a quasi-digital counter, has a finite number of microcells and this can give rise to a partial saturation of the output signal due to two or more photons interacting with the same microcell (multiple hit), simultaneously or during its recovery. This effect takes place massively with LaBr3 and CeBr3 scintillators, whose light emission produces a large number of photons in a very short time interval. This causes non-linearity when the deposited energy is small, but can almost blind the SiPM for a while in case of a large deposited energy in the small scintillator cube (see Figure 10, Figure 11, Figure 14 and Figure 17). The effect is more pronounced in the SiPM with a smaller number of microcells (Hamamatsu). The result of the above-mentioned interplay is summarized in Figure 19a, where the corresponding values of *R*^2^ indicate quite a poor linearity. In the bigger cylindrical configuration, there is a relevant advantage, i.e., the photons produced are shared among several SiPMs so that each one sees only a fraction of them. In such a case, the counting losses are strongly reduced, as can be seen in the worst-case plots of Figure 15, Figure 16 and Figure 18.

If one is interested in setting up a small scintillator with a single SiPM, the best candidate seems to be CsI(Tl), either using a SensL or a Hamamatsu SiPM. Indeed, CsI(Tl) represents the best trade-off between linearity, energy resolution (Table 4) and chemical properties:

- Detectors with LaBr3(Ce) and CeBr3, hygroscopic and thus requiring an expensive air-tight case, would be strongly non-linear as they tend to blind the SiPM;
- NaI(Tl) is nearly equivalent to CsI(Tl), but it is hygroscopic;
- BGO has a poor light yield; therefore, the SiPM light readout would be perfectly linear but would provide a poor energy resolution;
- CsI(Tl) is reasonably inexpensive compared to LaBr3(Ce), CeBr3 and NaI(Tl), and does not require any special air-tight case, thus being easy to manipulate.

Despite some claims about the possible non-linearity of similar SiPMs when detecting gamma rays above 1 MeV or less when coupled with CsI(Tl) [10], our calculations show no strong evidence of such an effect (Figure 17 and Figure 19a). Indeed, the experimental data in Figure 23 and Figure 24 hint at a slight loss of linearity above 1.7 MeV for both the SensL and the Hamamatsu SiPMs, but this can likely be ascribed to a non-linearity of the crystal itself, as was also suggested in [11].

We remark that the energy resolution values quoted in Table 4 do not consider corrections for the Fano factor, and they were simply calculated as a Poisson uncertainty from the number of triggered microcells (inverse square root multiplied by 2.35). However, they provide realistic indications, in particular for LaBr3(Ce) and CeBr3. In the same table, we also listed the expected position of the 662 keV peak for a ^137^Cs source in the energy scale obtained from the linear fit for the four studied cases. In order to highlight the non-linearity, in the table, we quoted the (1 − *R*^2^) value for each configuration.

**Figure 25:**
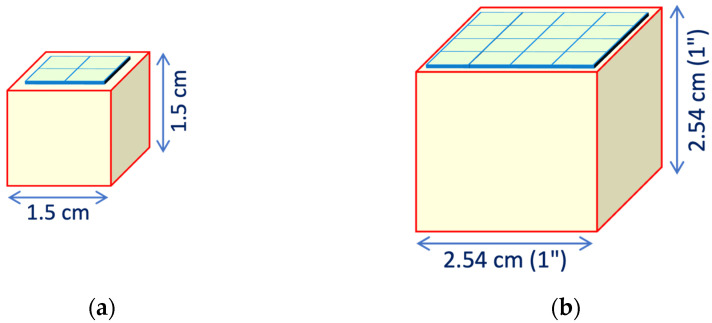
Two additional detector geometries evaluated: (**a**) 1.5 cm × 1.5 cm × 1.5 cm scintillator coupled to a 2 × 2 array of 6 mm × 6 mm SiPMs; (**b**) 2.54 cm × 2.54 cm × 2.54 cm scintillator coupled to a 4 × 4 array of 6 mm × 6 mm SiPMs.

**Figure 26:**
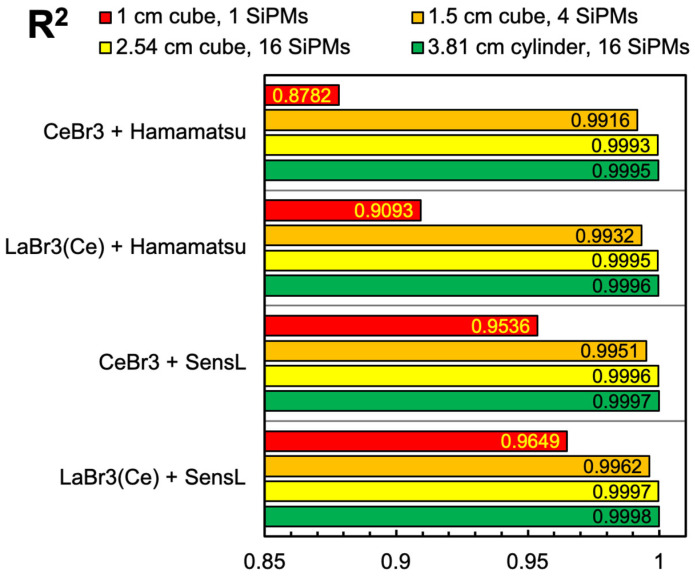
The *R*^2^ coefficient (Equation (6)), which quantifies the goodness of a linear fit, for four geometrical configurations of LaBr3(Ce) and CeBr3 scintillators read by different arrangements of SensL and Hamamatsu 6 mm × 6 mm SiPM arrays.

As a further exercise, we used our calculation method to investigate the behavior of bigger cubic geometries, only considering LaBr3(Ce) and CeBr3 scintillators because they are the only ones producing relevant non-linearity with the SiPM readout. Therefore, we examined the response as a function of the energy for the two additional cubic configurations of Figure 25. In Table 5, we summarize the main features of these configurations along with the previously shown cubic and cylindrical ones for comparison, and in Figure 26 we compare the corresponding results, which show an improving linearity trend as the crystal and the SiPM array sizes increase.

## 6. Conclusions

The results of our numerical analysis can likely be used when planning radiation detection systems based on scintillators and SiPMs, as it highlights their realistic linearity boundaries. The analysis also confirms the interesting properties of the CsI(Tl) scintillator, as it represents a good compromise between cost, performance and ease of use, especially if planning to employ it in miniature detection systems like personal dosimeters. For higher level spectroscopic applications of scintillators, the feasibility of larger cylindrical configurations, coupled to arrays of SiPMs, has been confirmed up to a few MeV. LaBr3(Ce) and CeBr3 are the best options in terms of timing and energy resolution, even though CsI(Tl) remains a notable candidate as well.

## Figures and Tables

**Table 3:** Energy values of the peaks used in the calibration for each source. The first two could not be used with the detector equipped with the SiPM because of electronic noise and the low activity of the ^226^Ra source. The two highest energy peaks from ^226^Ra, not used for the calibration, were used as a linearity cross-check.

Gamma Source	Peak Energy [keV]	Notes
^226^Ra	242	Only used with SensL
	295	
	352	SensL and Hamamatsu
	609	
	768	
	1120	
	1238	
	1377	
	1764	
	2202	Used for cross-check and not for calibration
	2448	
^137^Cs	662	SensL and Hamamatsu
^22^Na	511	SensL and Hamamatsu
	1274	
^60^Co	1173	SensL and Hamamatsu
	1330	

**Table 4:** Position of the 662 keV peak in the energy scale obtained via the linear fit, FWHM energy resolution at 662 keV and non-linearity up to 2.5 MeV for all the examined configurations. The value of (1 − *R*^2^) was chosen here to quantify the overall non-linearity. Notice that the resolution quoted here was naively calculated just for intercomparison as the inverse square root of the number of triggered microcells multiplied by 2.35.

		CsI(Tl)	LaBr3(Ce)	CeBr3	BGO	NaI(Tl)
SensL + cube	Efit @662 keV	664.7	715.4	726.4	663.3	671.2
	Resolution @662 keV	3.06%	2.89%	2.80%	7.20%	3.15%
	Non-linearity (1 − *R*^2^)	2.2 × 10^−4^	3.5 × 10^−2^	4.6 × 10^−2^	6.1 × 10^−5^	2.0 × 10^−3^
Hamamatsu + cube	Efit @662 keV	667.2	779.5	806.2	664.6	678.6
	Resolution @662 keV	2.50%	2.67%	2.63%	5.86%	2.74%
	Non-linearity (1 − *R*^2^)	7.3 × 10^−4^	9.1 × 10^−2^	1.2 × 10^−1^	2.2 × 10^−4^	4.9 × 10^−3^
SensL + cylinder	Efit @662 keV	662.2	664.5	664.8	662.1	662.6
	Resolution @662 keV	2.82%	2.45%	2.34%	6.65%	2.86%
	Non-linearity (1 − *R*^2^)	1.3 × 10^−6^	2.1 × 10^−4^	2.7 × 10^−4^	3.3 × 10^−7^	1.3 × 10^−5^
Hamamatsu + cylinder	Efit @662 keV	662.3	665.4	665.8	662.1	662.8
	Resolution @662 keV	2.29%	2.11%	2.04%	5.40%	2.44%
	Non-linearity (1 − *R*^2^)	2.6 × 10^−6^	3.8 × 10^−4^	4.6 × 10^−4^	7.0 × 10^−7^	2.2 × 10^−5^

**Table 5:** Summary of the main features of the evaluated detector configurations.

	Hamamatsu	SensL
Cube size	1 cm	
N. SiPMs	1	
N. microcells	14,331	18,980
Sensor/total area ratio	6%	
Collection efficiency	56%	
Cube size	1.5 cm	
N. SiPMs	4	
N. microcells	57,324	75,920
Sensor/total area ratio	10.7%	
Collection efficiency	70%	
Cube size	2.54 cm	
N. SiPMs	16	
N. microcells	229,296	303,680
Sensor/total area ratio	14.9%	
Collection efficiency	78%	
Cylinder size	3.81 cm	
N. SiPMs	16	
N. microcells	229,296	303,680
Sensor/total area ratio	8.4%	
Collection efficiency	65%	

## Data Availability

The data and the calculation workbooks used to produce them are available on request to the corresponding author.

## Appendix A

**Table A1:** Cube configuration: light collection efficiency results from Geant4 simulation and our simple model (these data are plotted in Figure 4 and Figure 5).

Reflectivity	Geant4	Simple Model	Simple/Geant4	Error
0.9	40.8%	39.0%	0.954	0.0047
0.91	43.6%	41.5%	0.952	0.0046
0.92	46.1%	44.4%	0.963	0.0045
0.93	49.1%	47.7%	0.971	0.0044
0.94	53.1%	51.5%	0.971	0.0042
0.95	57.9%	56.1%	0.969	0.0040
0.96	62.8%	61.5%	0.979	0.0039
0.97	69.4%	68.0%	0.980	0.0037
0.98	77.0%	76.1%	0.989	0.0036
0.99	87.0%	86.5%	0.994	0.0034
1	100.0%	100.0%	1.000	0.0032

**Table A2:** Cylinder configuration: light collection efficiency results from Geant4 simulation and our simple model (these data are plotted in Figure 4 and Figure 5).

Reflectivity	Geant4	Simple Model	Simple/Geant4	Error
0.9	49.5%	47.9%	0.967	0.0043
0.91	52.5%	50.5%	0.963	0.0042
0.92	55.4%	53.5%	0.966	0.0041
0.93	58.3%	56.8%	0.973	0.0040
0.94	62.0%	60.5%	0.976	0.0039
0.95	66.3%	64.8%	0.977	0.0038
0.96	71.2%	69.7%	0.979	0.0037
0.97	76.6%	75.4%	0.985	0.0036
0.98	83.1%	82.1%	0.989	0.0034
0.99	90.7%	90.2%	0.995	0.0033
1	100.0%	100.0%	1.000	0.0032

## Appendix B

**Table A3:** Number of triggered microcells as a function of deposited gamma energy for the cubic configuration of Figure 3a with the SensL SiPM (data plotted in Figure 17a).

Edep [keV]	CsI(Tl)	LaBr3(Ce)	CeBr3	BGO	NaI(Tl)
100	905	1178	1287	162	875
200	1806	2286	2488	324	1737
300	2702	3328	3608	485	2586
400	3594	4307	4652	646	3421
500	4482	5228	5626	807	4245
600	5365	6094	6534	967	5056
700	6245	6909	7382	1127	5856
800	7120	7676	8172	1286	6644
900	7991	8398	8910	1446	7420
1000	8859	9078	9598	1604	8186
1100	9722	9717	10,240	1762	8941
1200	10,581	10,320	10,838	1920	9685
1300	11,436	10,887	11,397	2078	10,419
1400	12,288	11,422	11,918	2235	11,144
1500	13,135	11,926	12,405	2392	11,858
1600	13,979	12,401	12,859	2548	12,563
1700	14,819	12,849	13,283	2704	13,259
1800	15,655	13,271	13,678	2860	13,946
1900	16,487	13,670	14,048	3015	14,623
2000	17,316	14,046	14,392	3170	15,292
2100	18,141	14,402	14,714	3324	15,953
2200	18,962	14,737	15,015	3478	16,605
2300	19,780	15,055	15,296	3632	17,249
2400	20,594	15,354	15,558	3786	17,885
2500	21,404	15,638	15,803	3939	18,514

**Table A4:** Number of triggered microcells as a function of deposited gamma energy for the cubic configuration of Figure 3a with the Hamamatsu SiPM (data plotted in Figure 17b).

Edep [keV]	CsI(Tl)	LaBr3(Ce)	CeBr3	BGO	NaI(Tl)
100	1371	1559	1661	246	1193
200	2730	2955	3130	490	2355
300	4076	4207	4431	734	3487
400	5410	5330	5583	976	4590
500	6732	6338	6603	1218	5665
600	8043	7243	7507	1458	6714
700	9342	8056	8307	1697	7737
800	10,630	8788	9016	1934	8737
900	11,906	9447	9645	2171	9713
1000	13,172	10,042	10,202	2407	10,666
1100	14,426	10,578	10,697	2641	11,598
1200	15,670	11,063	11,135	2875	12,510
1300	16,904	11,501	11,524	3107	13,402
1400	18,127	11,899	11,870	3339	14,274
1500	19,341	12,259	12,177	3569	15,128
1600	20,544	12,587	12,450	3798	15,965
1700	21,738	12,886	12,693	4026	16,784
1800	22,921	13,159	12,909	4254	17,588
1900	24,096	13,409	13,102	4480	18,375
2000	25,261	13,637	13,274	4705	19,147
2100	26,417	13,847	13,427	4929	19,904
2200	27,563	14,041	13,565	5152	20,647
2300	28,701	14,220	13,687	5375	21,377
2400	29,830	14,386	13,797	5596	22,093
2500	30,951	14,539	13,896	5816	22,796

**Table A5:** Number of triggered microcells as a function of deposited gamma energy for the cylindrical configuration of Figure 3b with the SensL SiPM (data plotted in Figure 18a).

Edep [keV]	CsI(Tl)	LaBr3(Ce)	CeBr3	BGO	NaI(Tl)
100	1053	1407	1543	188	1023
200	2105	2808	3078	377	2045
300	3157	4203	4606	565	3066
400	4209	5592	6126	754	4086
500	5260	6974	7638	942	5104
600	6311	8350	9142	1130	6122
700	7362	9720	10,639	1319	7138
800	8412	11,084	12,129	1507	8153
900	9462	12,441	13,611	1695	9167
1000	10,511	13,793	15,085	1883	10,180
1100	11,560	15,139	16,552	2071	11,191
1200	12,609	16,478	18,011	2260	12,202
1300	13,657	17,812	19,463	2448	13,211
1400	14,705	19,139	20,908	2636	14,220
1500	15,753	20,461	22,346	2824	15,227
1600	16,800	21,777	23,776	3012	16,233
1700	17,847	23,086	25,199	3200	17,238
1800	18,894	24,390	26,615	3388	18,242
1900	19,940	25,689	28,023	3575	19,244
2000	20,986	26,981	29,425	3763	20,246
2100	22,031	28,267	30,820	3951	21,246
2200	23,076	29,548	32,207	4139	22,245
2300	24,121	30,823	33,587	4327	23,244
2400	25,165	32,093	34,961	4514	24,241
2500	26,209	33,357	36,328	4702	25,237

**Table A6:** Number of triggered microcells as a function of deposited gamma energy for the cylindrical configuration of Figure 3b with the SensL SiPM (data plotted in Figure 18b).

Edep [keV]	CsI(Tl)	LaBr3(Ce)	CeBr3	BGO	NaI(Tl)
100	1599	1905	2041	286	1403
200	3196	3799	4068	572	2804
300	4793	5681	6081	858	4203
400	6389	7552	8081	1143	5599
500	7985	9412	10,068	1429	6994
600	9579	11,260	12,042	1715	8386
700	11,173	13,098	14,002	2000	9777
800	12,766	14,924	15,949	2286	11,165
900	14,358	16,739	17,884	2571	12,552
1000	15,949	18,544	19,805	2856	13,936
1100	17,539	20,337	21,714	3141	15,318
1200	19,129	22,120	23,610	3427	16,699
1300	20,718	23,892	25,493	3712	18,077
1400	22,306	25,654	27,364	3997	19,453
1500	23,893	27,404	29,222	4282	20,827
1600	25,480	29,145	31,068	4566	22,199
1700	27,065	30,874	32,901	4851	23,569
1800	28,650	32,594	34,723	5136	24,937
1900	30,234	34,303	36,532	5421	26,303
2000	31,817	36,002	38,329	5705	27,668
2100	33,400	37,690	40,114	5990	29,030
2200	34,982	39,369	41,888	6274	30,390
2300	36,562	41,037	43,649	6558	31,748
2400	38,142	42,696	45,399	6843	33,104
2500	39,722	44,344	47,137	7127	34,458
